# Characterization of a P1-bacteriophage-like plasmid (phage-plasmid) harbouring *bla*_CTX-M-15_ in *Salmonella enterica* serovar Typhi

**DOI:** 10.1099/mgen.0.000913

**Published:** 2022-12-05

**Authors:** David R. Greig, Matthew T. Bird, Marie Anne Chattaway, Gemma C. Langridge, Emma V. Waters, Paolo Ribeca, Claire Jenkins, Satheesh Nair

**Affiliations:** ^1^​ National Infection Service, UK Health Security Agency, London NW9 5EQ, UK; ^2^​ NIHR Health Protection Research Unit in Gastrointestinal Pathogens, Liverpool, UK; ^3^​ Division of Infection and Immunity, Roslin Institute and Royal (Dick) School of Veterinary Studies, University of Edinburgh, Easter Bush EH25 9RG, UK; ^4^​ NIHR Health Protection Research Unit in Healthcare Associated Infections and Antimicrobial Resistance, Oxford, UK; ^5^​ Quadram Institute Bioscience, Norwich Research Park, Norwich, UK; ^6^​ NIHR Health Protection Research Unit in Genomics and Enabling Data, Warwick, UK

**Keywords:** bacteriophage, Illumina, Nanopore, phage-plasmid, plasmid, *Salmonella enterica* serovar Typhi

## Abstract

Antimicrobial-resistance (AMR) genes can be transferred between microbial cells via horizontal gene transfer (HGT), which involves mobile and integrative elements such as plasmids, bacteriophages, transposons, integrons and pathogenicity islands. Bacteriophages are found in abundance in the microbial world, but their role in virulence and AMR has not fully been elucidated in the *Enterobacterales *. With short-read sequencing paving the way to systematic high-throughput AMR gene detection, long-read sequencing technologies now enable us to establish how such genes are structurally connected into meaningful genomic units, raising questions about how they might cooperate to achieve their biological function. Here, we describe a novel ~98 kbp circular P1-bacteriophage-like plasmid termed ph681355 isolated from a clinical *Salmonella enterica* serovar Typhi isolate. It carries *bla*_CTX-M-15_, an IncY plasmid replicon (*repY* gene) and the IS*EcP1* mobile element and is, to our knowledge, the first reported P1-bacteriophage-like plasmid (phage-plasmid) in *S *. *enterica* Typhi. We compared ph681355 to two previously described phage-plasmids, pSJ46 from *S *. *enterica* serovar Indiana and pMCR-1-P3 from *Escherichia coli *, and found high nucleotide similarity across the backbone. However, we saw low ph681355 backbone similarity to plasmid p60006 associated with the extensively drug-resistant *S *. *enterica* Typhi outbreak isolate in Pakistan, providing evidence of an alternative route for *bla*_CTX-M-15_ transmission. Our discovery highlights the importance of utilizing long-read sequencing in interrogating bacterial genomic architecture to fully understand AMR mechanisms and their clinical relevance. It also raises questions regarding how widespread bacteriophage-mediated HGT might be, suggesting that the resulting genomic plasticity might be higher than previously thought.

## Data Summary

All fastq files and assemblies were submitted to the National Center for Biotechnology Information (NCBI). All data can be found under BioProject PRJNA248792 – https://www.ncbi.nlm.nih.gov/bioproject/PRJNA248792. Strain-specific details can be found in Methods under data deposition.

**Impact Statement**

Whole-genome sequencing has revolutionized the way we identify and characterize antimicrobial-resistance (AMR) genes and regions/elements in pathogens. Illumina sequencing coupled with Nanopore sequencing and careful data curation allows the mining of pathogen genomes to detect, characterize and track novel mobile elements involved in AMR transmission. In this study, we have identified a circular P1-bacteriophage-like plasmid (termed phage-plasmid) harbouring a *bla*_CTX-M-15_ gene conferring extended-spectrum β-lactamase resistance in *Salmonella enterica* serovar Typhi. It is the first time, to our knowledge, that such a DNA element has been described in this organism. There is increasing evidence from the literature to show that the horizontal spread of AMR genes mediated by bacteriophages and bacteriophage-like plasmid elements is much more common than previously envisioned. This current study shows the potential ability of using Nanopore sequencing for the detection and characterization of these elements, highlighting the importance of including long-read sequence data for the screening and surveillance of mechanisms involved in AMR transmission. Understanding AMR carriage and transmission patterns provides information to support appropriate clinical management and inform implementation of public-health control measures.

## Introduction

*Salmonella enterica* subspecies *enterica* serovar Typhi is the causative agent of typhoid fever and is associated with an estimated 11 million infections and 116 000 deaths globally each year [1]. The majority of this disease burden is concentrated in South Asia and other low to middle income countries (LMICs) [1].

Growing rates and spread of antimicrobial resistance (AMR) pose a threat to the effective empirical treatment and control of typhoid fever. Third-generation cephalosporins (extended spectrum β-lactams) are frequently used in the treatment of typhoid fever. The emergence and spread of extensively drug-resistant (XDR) *S *. *enterica* Typhi in Pakistan, and now globally, is a public-health concern as it has left azithromycin (one of the last oral antibiotics available) and meropenem (carbapenem) as the only options available for treatment [2–8] (https://emergency.cdc.gov/han/2021/han00439.asp).

Resistance to cephalosporins is principally mediated by acquisition of a plasmid carrying extended-spectrum β-lactamase (ESBL) genes, the most prevalent of which are the CTX-M type ESBLs [9]. Recently *bla*_CTX-M-15_ and *bla*_CTX-M-55_ present on a transmissible ~84 kbp IncY incompatibility group plasmid (p60006) were shown to be associated with extended-spectrum β-lactam resistance in XDR outbreak *S *. *enterica* Typhi isolates from Pakistan [2, 3]. We are also now observing chromosomal integration of ESBL genes and the loss of the IncY plasmid in these XDR *S *. *enterica* Typhi isolates, demonstrating evolution of the AMR drug region throughout the outbreak [3].

Mobile and integrative genetic elements, including plasmids, bacteriophages, transposons, integrons and pathogenic islands, are important vehicles of horizontal gene transfer (HGT) enabling transmission of genetic information between bacteria [10]. Plasmids are considered the most common and important genetic element to spread ESBLs between bacterial strains, but studies have shown the mobilization or transfer of AMR genes by bacteriophages in various bacterial species, including *Escherichia coli* and non-typhoidal *Salmonella* (NTS) [11–15], and reported a *mcr*-1 gene in *E. coli* and a *bla*_CTX-M-27_ gene in NTS present on bacteriophage-like IncY elements (phage-plasmid) that were 97 and 104 kbp in size, respectively. In another report [13], a 115 kbp circular P1-like bacteriophage harbouring a *bla*_SHV-12_ element in *E. coli* was characterized. P1-like bacteriophages are known to replicate in their host as independent low copy number plasmid-like elements [13].

Recent advances in sequencing technologies, especially long-read sequencing, now enable us to identify and characterize novel bacteriophage/plasmid-like elements, as well as look at their genetic diversity [16–18]. Long-read sequencing has also enabled the ability to characterize the genetic architecture of individual mobile integrative elements harbouring AMR determinants [19–23], which is essential in order to characterize the precise biological mechanisms underpinning HGT mechanisms.

In this study, we describe a circular P1-bacteriophage-like plasmid (phage-plasmid) harbouring a *bla*_CTX-M-15_ gene and IncY plasmid replicon (*repY*) isolated from a clinical *S *. *enterica* Typhi isolate from a traveller returning to the UK from Iraq. To the best of our knowledge, this is the first time such a genomic element, and the implications of its presence towards AMR acquisition and maintenance, have been described for *S *. *enterica* Typhi. That is especially relevant due to the current heavy burden of the disease, and the pathogenic potential that *S *. *enterica* Typhi strains carrying AMR might present in the future.

## Methods

### Strain selection and details

A laboratory-confirmed *S *. *enterica* Typhi isolate termed 681 355 was referred to the Gastrointestinal Bacterial Reference Unit (GBRU), UK Health Security Agency (UKHSA) [formally Public Health England (PHE)] in January 2019. This isolate was from a traveller returning to the UK from Iraq. Epidemiological information and phylogenetic analysis of this isolate have previously been described by Godbole *et al*. [24]. Ethical approval for the detection of gastrointestinal bacterial pathogens from faecal specimens, or the identification, characterization and typing of cultures of gastrointestinal pathogens, submitted to GBRU is not required as it is covered by UKHSA’s surveillance mandate.

### Antimicrobial-susceptibility testing

Antimicrobial-susceptibility testing was performed on this isolate as described by Chattaway *et al*. [25]. Minimum inhibitory concentrations (MICs) were determined by agar dilution using Mueller–Hinton agar for the standard panel of antibiotics recommended for *Salmonella* spp. by the European Committee on Antimicrobial Susceptibility Testing (EUCAST); breakpoints and screening concentration criteria were used for interpretation of results as described by EUCAST (2020; https://www.eucast.org/).

### DNA extraction, library preparation, Illumina sequencing and data processing

Genomic DNA was extracted from *S *. *enterica* Typhi culture using the QIAsymphony system (Qiagen). The sequencing library was prepared using the Nextera XP kit (Illumina) for sequencing on the HiSeq 2500 instrument (Illumina), run with the fast protocol. fastq reads were processed using Trimmomatic v0.27 [26] to remove bases with a Phred score of <30 from the leading and trailing ends, with reads <50 bp after quality trimming discarded.

### Genotyping and *in silico* AMR typing

Sequence type (ST) and serovar were determined from reads using most (v1.0) as described by Tewolde *et al*. [27] and eBURST group (eBG) as described by Achtman *et al*. [28].

Resistance genes for the *S *. *enterica* Typhi isolate used in the study were detected using GeneFinder (https://github.com/phe-bioinformatics/gene_finder), a customized algorithm that uses Bowtie2 (v2.3.5.1) [29] to align reads to a set of reference sequences, and SAMtools (v1.8) [30], to generate an mpileup file, as previously described [31]. Briefly, the data are parsed based on read coverage of the query sequence (100 %), consensus base-call on variation (>85 %) and the nucleotide identity (>90 %) to determine the presence of the reference sequence or nucleotide variation within that sequence. β-Lactamase variants were determined with 100 % identity using the reference sequences downloaded from ResFinder [32] or the National Center for Biotechnology Information (NCBI) β-lactamase data resources (https://www.ncbi.nlm.nih.gov/pathogens/beta-lactamase-data-resources). Known acquired resistance genes and resistance-conferring mutations relevant to β-lactams, fluroquinolones, aminoglycosides, chloramphenicol, macrolides, sulphonamides, tetracyclines, trimethoprim, rifamycins and fosfomycin were included in the analysis [33, 34].

### DNA extraction, library preparation, Nanopore sequencing and data processing

High-molecular mass DNA was extracted from *S *. *enterica* Typhi isolate 681 355 using the Fire Monkey HMW DNA extraction kit (RevoluGen) and DNA concentration was determined via Qubit (Thermofisher Scientific), as previously described [23]. Library preparation was performed using the rapid barcoding kit (SQK-RBK004) (Oxford Nanopore Technologies). The prepared library was loaded onto a FLO-MIN106 R9.4.1 flow cell (Oxford Nanopore Technologies) and sequenced using the MinION system (Oxford Nanopore Technologies) for 72 h.

Data produced in a raw FAST5 format was basecalled using Guppy v3.2.6 Fast model (Oxford Nanopore Technologies) into fastq format. Read de-multiplexing, quality control, trimming and filtering were completed as described elsewhere [35] with the only modification being bases=490 Mbp, to generate approximately 100× coverage of a *Salmonella* genome (approximately 4.9 Mbp).

### *De novo* assembly, correction, re-orientation and annotation

The filtered Nanopore fastq file with the 100× coverage of longest reads was assembled using Flye v2.8 [36] with default parameters enabled. Correction (polishing) of the assembly was performed in a modified two-step process described previously [35, 37]. Firstly, Pilon v1.22 [38] was used with Illumina fastq reads as the query dataset with the use of bwa v0.7.17 [39] and SAMtools v1.7 [30]. Secondly, Racon v1.3.3, [40] also using bwa v0.7.17 [39] and SAMtools v1.7 [30], was used again with the Illumina fastq reads. As the chromosome was circular and closed, it was re-orientated to start at the *dnaA* gene (GenBank accession no. NC_000913) from *E. coli* K-12, using the --fixstart parameter in Circlator v1.5.5 [41]. Prokka v1.13 [42] was used to annotate the final assembly.

### *In silico* plasmid typing and comparison of ph681355 and replicon to publicly available sequences

The plasmid replicon was identified for each non-chromosomal contig within the final assembly using PlasmidFinder v2.1 [43] with the *Enterobacteriaceae *, minimum identity=90 % and minimum coverage=90 % parameters set. brig [44] was used to compare ph681355 to the *bla*_CTX-M-27_ *S *. *enterica* serovar Indiana Chinese SJ46 phage-plasmid (GenBank accession no. NC_031129), *E. coli* bacteriophage P1 (accession no. AF234172), the *mcr*-1 *E. coli* phage-plasmid (accession no. KX880944) and plasmid p60006 (accession no. LT906492) isolated from a Pakistan XDR *S *. *enterica* Typhi outbreak isolate that also harboured *bla*_CTX-M-15_. Parameters used included -perc_identity=90 and -*e* value=1×10-10. The coding sequences (CDSs) were annotated using Prokka v1.13 [42] as stated in the previous section, with AF234172 acting as a reference for CDS and gene annotation. The *repY* genes from the above plasmids were compared to the *repY* gene from ph681355 also using blastn [45].

### Detection and characterization of ph681355 structural variation

To determine whether multiple isoforms were present within the Nanopore reads, the Nanopore fastq reads for the sample were aligned to the finalized assembly using Minimap2 v2.17 [46] and SAMtools v0.7.17 [30]. The alignment was visualized using Integrative Genomics Viewer (igv) v2.12.3 [47] and the breakpoints of each isoform were identified. Once breakpoints were identified relative to each isoform, those positions were used with SAMtools v0.7.17 [30] to isolate reads that aligned and spanned across both ends of each breakpoint (i.e. spanned the homologous region in question). Any reads that aligned across a given set of breakpoints had to share the same size as it existed in the fastq file, and not be clipped within the alignment, to be considered. From here, the relative proportions of reads aligning to each isoform were calculated.

### Data deposition

Illumina and Nanopore fastq files and polished assembly for *S *. *enterica* Typhi isolate 681 355 are available from the NCBI under BioProject PRJNA248792. The SRA (sequence read archive) accession numbers are as follows: Illumina fastq – SRR8554071; Nanopore fastq – SRR16296518. The GenBank accession numbers are CP083411 for the chromosome and CP083412 for ph681355 (phage-plasmid).

## Results

### Sample 681355 genome statistics and genotyping

Isolate 681 355 was confirmed to be *S *. *enterica* Typhi ST1, a member of serovar Typhi eBURST group 13 (eBG13). Previous phylogenetic analysis confirmed that this strain sat within the dominant global H58 haplotype, but it did not cluster with the recent XDR *S *. *enterica* Typhi outbreak strains in Pakistan [2, 24]. Nanopore sequencing and processing produced a final genome of two contigs, one chromosome (4 782 729 kbp) and one of 98 174 kbp (ph681355).

### Phenotypic and genotypic resistance to extended-spectrum β-lactam

*S *. *enterica* Typhi isolate 681 355 was found to have minimum inhibitory concentrations (MICs) to the following antimicrobials (in µg µl^−1^): amoxicillin [>128 (R)], ciprofloxacin [0.06 (R)], ceftriaxone [>64 (R)], cotrimoxazole [1 (S)], ertapenem [0.25 (S)] and azithromycin [<2 (S)] as observed by Chattaway *et al*. [25]. Genotypic mapping of AMR determinants did not reveal the presence of carbapenem, fosfomycin and azithromycin resistance, but showed the presence of *bla*_CTX-M-15_, a point mutation in *gyrA* [83:S-F] and IncY plasmid replicon (*repY*) genes.

### Characterization and comparison of ph681355

**Fig. 1.**
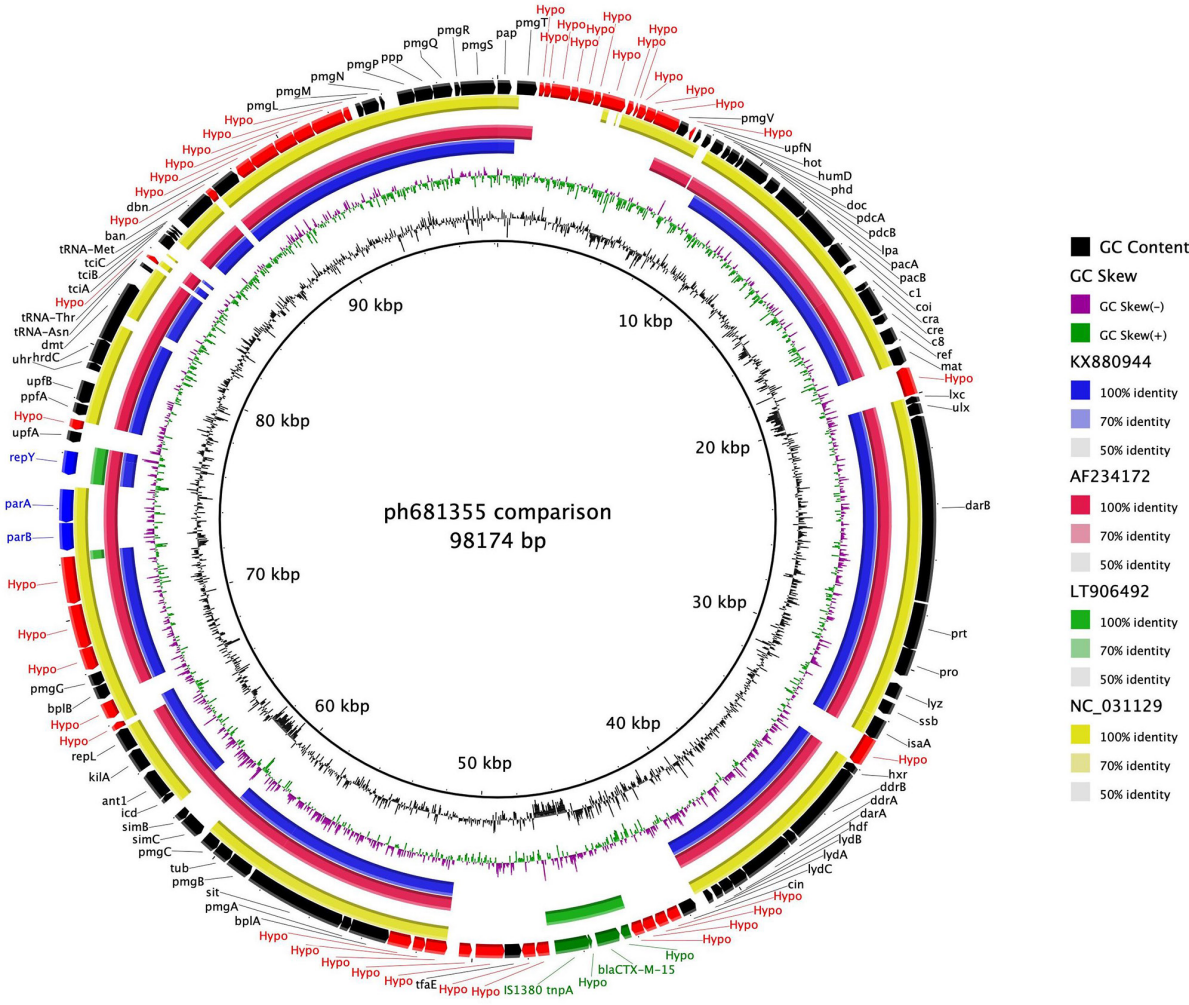
brig plot of ph681355 as the reference versus PMCR-1-P3 phage-plasmid shown as the blue ring (GenBank accession no. KX880944) – *E coli* plasmid; bacteriophage P1 as the red ring (accession no. AF234172); plasmid p60006 as the green ring (accession no. LT906492) – *S *. *enterica* Typhi; and finally SJ46 phage-plasmid (accession no. NC_031129) shown as the yellow ring. Also shown are CDSs (genes) on the outer ring, with green showing AMR cassette, blue showing plasmid maintenance genes, black showing bacteriophage-associated genes and red showing hypothetical proteins.

ph681355 is ~98 kbp in size. The ph681355 contig was annotated to contain 118 CDSs, of which 8 (7 %) are maintenance genes (Fig. 1). A 3.5 kbp IS*Ecpl-bla*_CTX-M-15_-*tnpA* gene resistance cassette was confirmed to be present on ph681355. Plasmid typing using PlasmidFinder determined the replicon type to be IncY plasmid (*repY*) (Fig. 1). The *repY* gene from ph681355 matched bacteriophage P1 (GenBank accession no. AF234172), phage-plasmid PMCR-1-P3 (accession no. KX880944) and plasmid p60006 (accession no. LT906492) *repY* genes 100, 88 and 100 % at the nucleotide level, respectively. Notably, *repY* is absent from phage-plasmid SJ46 (NC_031129) from the *S *. enterica* s*erovar Indiana isolate from China.

Sequence analysis of ph681355 compared to publicly available P1 bacteriophage from *E. coli* (AF234172), phage-plasmids SJ46 from *S *. enterica* s*erovar Indiana (NC_031129) and PMCR-1-P3 from *E. coli* (KX880944) showed 79.7, 78.0 and 78.5% nucleotide similarity, respectively, across the sequenced region (Fig. 1). Hence, ph681355 (like SJ46 and PMCR-1-P3) was characterized as a chimeric element termed as phage-plasmid (phage and bacterial genes present). The main region of variation was the absence of the 3.5 kbp IS*Ecpl-bla*_CTX-M-15_-*tnpA* resistance gene cassette in bacteriophage P1, phage-plasmids SJ46 and PMCR-1-P3 (Fig. 1).

There was only a 4.9 % sequence similarity between ~98 kbp phage-plasmid ph681355 and the ~84 kbp plasmid p60006 (LT906492), although the *repY* plasmid replicon gene and IS*Ecpl*-*bla*_CTX-M-15_-*tnpA* resistance cassette were present in both (Fig. 1). Our analysis shows that the ESBL resistance is carried by the same 3.5 kbp IS*Ecpl-bla*_CTX-M-15_-*tnpA* resistance gene cassette but that different mechanisms are involved in the transmission of ESBL resistance.

### Detection and characterization of structural variation on ph681355

**Fig. 2.**
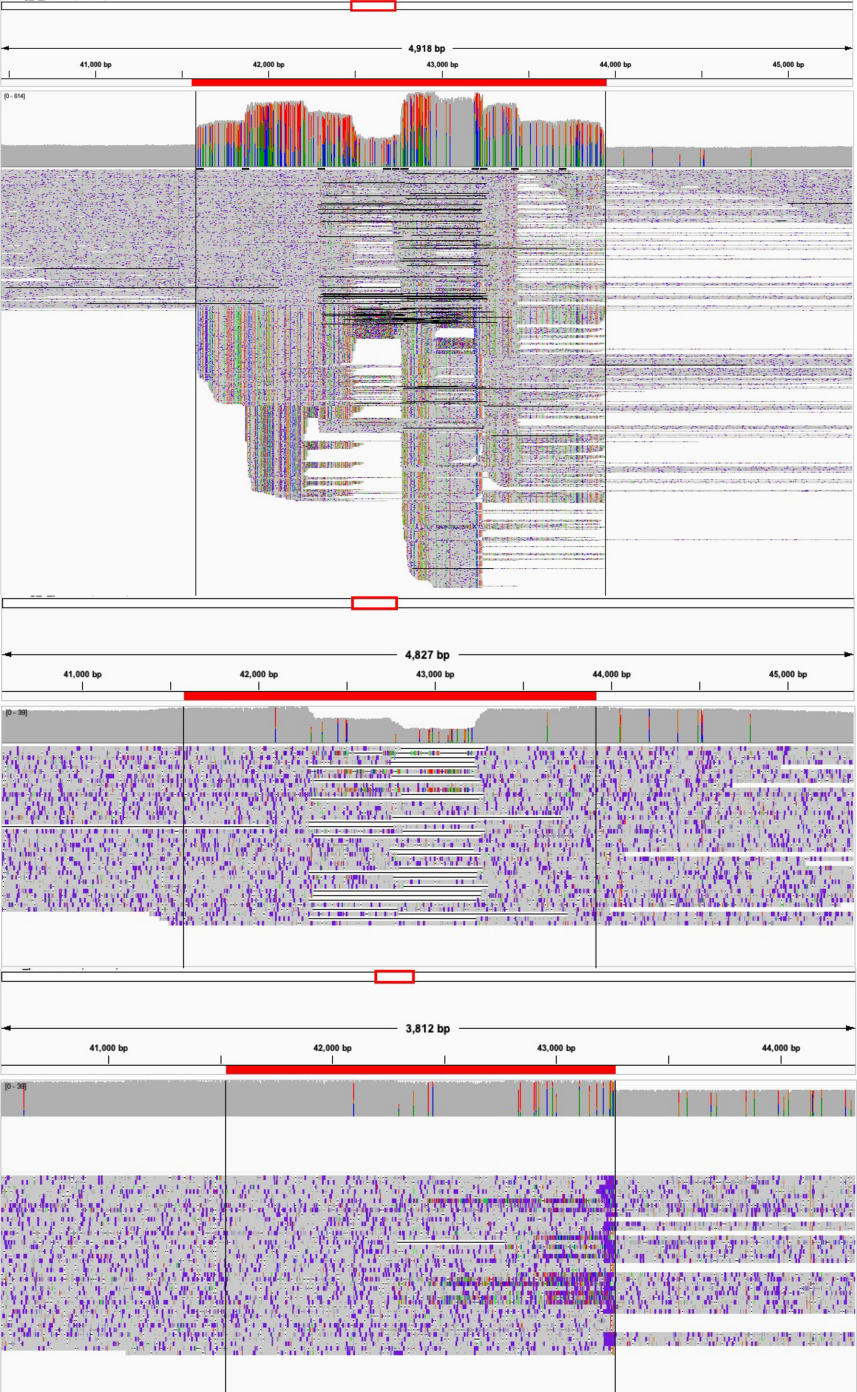
Integrative Genomics Viewer (igv) visualization of the alignment of reads for the region of ph681355 where the *bla*_CTX-M-15_ is located (top). Also showing the same alignment with only reads spanning across the *bla*_CTX-M-15_ mobile genetic element (middle). Finally, showing the alignment of reads with the *bla*_CTX-M-15_ mobile genetic element removed *in silico* (bottom). The red line above each part of the figure (top, middle, bottom) indicates the *tnpA*-Hypo-*bla*-Hypo element.

When confirming the validity of the ph681355 contig, it was noted that some Nanopore reads were clipped in the alignment at the same loci as the mobile genetic element in which the *bla*_CTX-M-15_ gene is located (Fig. 2). Artificial (*in silico*) removal of the *bla*_CTX-M-15_ mobile genetic element also showed Nanopore reads aligning across this region with the absence of clipping (Fig. 2). This suggests that there are two isoforms of the ph681355 within the single Nanopore read set, one set (approximately 40 % of reads) confirming the presence of the mobile genetic element within ph681355 and a second set (approximately 60 %) aligning correctly (with no clipping) suggesting the absence of the mobile genetic element from ph681355.

## Discussion

The transmission of ESBL resistance is increasing in *Enterobacterales* mainly due to the presence of *bla*_CTX-M_ and *bla*_SHV_ class genes [3, 13–15, 48, 49], and highlighted by the recent XDR *S *. *enterica* Typhi outbreak isolates in Pakistan that have subsequently spread globally [2, 3, 25, 50]. The *bla*_CTX-M-15_ gene responsible for ESBL resistance was present on a ~84 kbp IncY plasmid (p60006). Nair *et al*. [3] showed the rapid evolution of *bla*_CTX-M-15_ resistance mechanisms with the loss of the plasmid and the integration of *bla*_CTX-M-15_ into various regions of the chromosome of XDR *S *. *enterica* Typhi isolates.

In this study, a *S *. *enterica* Typhi isolate (681355) from a patient returning from Iraq had a *bla*_CTX-M-15_ gene present on an extrachromosomal element harbouring a plasmid replication *repY* gene. Phylogenetic analysis conducted by Godbole *et al*. [24] showed that this Iraqi *S *. *enterica* Typhi strain belonged to the global H58 haplotype but did not cluster with the XDR *S *. *enterica* Typhi outbreak Pakistani isolates [2].

Using Nanopore sequencing data, we identified a ~98 kbp extrachromosomal element (ph681355) that was ~14 kbp larger than plasmid p60006 described in the XDR *S *. *enterica* Typhi outbreak isolates from Pakistan [2]. ph681355 shared only the *repY* gene and the 3.5 kbp IS*Ecpl*-*bla*_CTX-M-15_-*tnpA* gene cassette with p60006 (Fig. 1).

Multiple modes for the rapid transmission of AMR and virulence genes involving mobile genetic elements have been widely described [51, 52]. More recently, studies have used long-read sequence data to demonstrate the involvement of P1- and P7-like bacteriophage elements in AMR gene transfer [13–15]. These bacteriophage-like elements (phage-plasmid) have both the bacteriophage-like lytic replication (*repL*) and plasmid replication (*rep*) genes, which we observed in ph681355 [13–15] (Fig. 1).

However, a phage-plasmid had only been described once in *S *. enterica* s*erovar Indiana [14] and, hence, to our knowledge ph681355 is the first one described in *S *. *enterica* Typhi. ph681355 is a chimeric molecule that consists of a bacteriophage backbone, a plasmid replicon (*rep* gene), a drug-resistance region(s)/gene(s) (e.g. a IS*Ecpl-bla*_CTX-M-15_-*tnpA* gene cassette) and a lysogenized P1 bacteriophage sequence [49] that have resulted from recombination of integrative elements on plasmids and prophage (chromosomally integrated, lysogenized bacteriophages) genomes.

Our findings provide supporting increasing evidence of the role played by viral vectors in the vertical and horizontal transfer of AMR genes and mobile elements between bacteria [13–15, 49]. Phage-plasmids of sizes between 90 and 120 kbp have been described in other *Enterobacterales *, such as *Citrobacter *, *Enterobacter* and *Pantoea* [17], and it has been suggested that the *repY* replicon in both plasmids and phage-plasmids is associated with strains harbouring ESBL genes [13, 53]. However, the *bla*_CTX-M-27_ gene in the *S *. enterica* s*erovar Indiana isolate [14] was present on a 104 kbp *repA*-like phage-plasmid and a *bla*_CTX-M-15_ gene was present on a 94 kbp IncF1A phage-plasmid in *Klebsiella pneumoniae* [49]. Phage-plasmids of different compatibility groups can be involved in the transmission of additional resistance determinants such as colistin (*mcr*-1) [15].

Nanopore sequencing provided extra context, revealing two isoforms of the same phage-plasmid structure, with and without a *bla*_CTX-M-15_ mobile genetic element (Fig. 2). Our observation suggests an ongoing process, whereby AMR genes can be dynamically acquired and lost depending on the evolutionary pressures surrounding the phage-plasmid and its host.

We are beginning to detect previously undescribed elements of AMR transmission, such as phage-plasmids, due to our ability to assemble complete genomes via long-read sequencing. Understanding the structure of such genomic elements is essential to fully elucidate the biological mechanisms of AMR and their clinical relevance. For instance, with long-read sequencing data we are now able to detect structural variants within the reads of a single bacterial culture [54]. This is showcased by our observation that in a proportion of the culture the *bla*_CTX-M-15_ resistance cassette is missing from the phage-plasmid (Fig. 2).

However, technical difficulties arise from genome assembly, which at times requires manual curation to ensure the validity of these assemblies for downstream analyses [37, 55, 56]. The same is true for accurate prophage and phage-plasmid detection and annotation directly from assemblies. Even from the abundant short-read sequence data (e.g. Illumina) and rapidly increasing long-read data, prophages and phage-plasmids sequences can often be missed [56, 57]. This is exacerbated by the number of *in silico* bacteriophage detection tools, which produce differing results from methodologies varying from reference-based detection through to machine-learning approaches [56, 58–63].

In conclusion, this study demonstrates the use of modern sequencing and data curation techniques in the successful detection, characterization and tracking of novel mobile elements involved in AMR transmission. We show the potential role of phage-plasmids in the capture and spread of ESBL resistance genes in *S *. *enterica* Typhi, in agreement with an increasing body of evidence showing the importance of horizontal spread of AMR genes mediated by bacteriophages and phage-plasmids [13]. Our findings also demonstrate the utility of long-read sequencing for non-typeable plasmids and phage-plasmids in detection and surveillance procedures once adequate sequencing capabilities become available. Future work will include the screening of gastrointestinal bacterial pathogens at UKHSA for the presence of *repL* and other relevant genes to detect the involvement of phage-plasmids in the transmission of AMR genes.
